# LncRNA TUG1 sponges miR-145 to promote cancer progression and regulate glutamine metabolism via Sirt3/GDH axis

**DOI:** 10.18632/oncotarget.21922

**Published:** 2017-10-19

**Authors:** Bing Zeng, Huilin Ye, Jianming Chen, Di Cheng, Canfeng Cai, Guoxing Chen, Xiang Chen, Haiyang Xin, Chaoming Tang, Jun Zeng

**Affiliations:** ^1^ Department of Hepatopancreatobiliary Surgery, Sun Yat-sen Memorial Hospital of Sun Yat-sen University, Guangzhou 510120, China; ^2^ Department of Gastrointestinal Surgery, The Sixth Affiliated Hospital of Guangzhou Medical University, Qing Yuan People’s Hospital, Guangdong 511518, China; ^3^ Department of Gastroenterology, The 251st Hospital of PLA, Zhangjiakou 075000, China; ^4^ Department of Gastroenterology, Sun Yat-sen Memorial Hospital of Sun Yat-sen University, Guangzhou 510120, China

**Keywords:** TUG1, intrahepatic cholangiocarcinoma, miR-145, glutamine metabolism, Sirt3

## Abstract

Long noncoding RNAs (lncRNAs) are important regulators in cancer progression. Deregulation of the lncRNA taurine upregulated gene 1 (TUG1) predicts poor prognosis and is implicated in the development of several cancers. In this study, we investigated the role of TUG1 in the pathogenesis of intrahepatic cholangiocarcinoma (ICC). We found that TUG1 is upregulated in ICC samples, which correlates with poor prognosis and adverse clinical pathological characteristics. Knockdown of TUG1 inhibited the proliferation, motility, and invasiveness of cultured ICC cells, and decreased tumor burden in a xenograft mouse model. When we explored the mechanisms underlying these effects, we found that TUG1 acts as an endogenous competing RNA (ceRNA) that ‘sponges’ miR-145, thereby preventing the degradation of Sirt3 mRNA and increasing expression of Sirt3 and GDH proteins. Accordingly, glutamine consumption, α-KG production, and ATP levels were dramatically decreased by TUG1 knockdown in ICC cells, and this effect was reversed by miR-145 inhibition. These findings indicate that the TUG1/miR-145/Sirt3/GDH regulatory network may provide a novel therapeutic strategy for treatment of ICC.

## INTRODUCTION

Intrahepatic cholangiocarcinoma (ICC) arises from the intrahepatic biliary tract and is the second most common primary liver malignant neoplasm. Because of lack of effective treatments besides radical resection, late clinical presentation, and rapid turnover, the prognosis of ICC is very poor [1]. Therefore, biomarkers that can predict tumor progression and prognosis would be an important decision-making tool. Unfortunately, the mechanisms underlying ICC pathogenesis are largely unknown.

Long noncoding RNAs (lncRNAs) are a heterogeneous class of long transcripts (> 200 nucleotides) that have a wide range of biological functions [2]. A growing number of studies demonstrated that lncRNAs are key regulators of tumor growth [3, 4]. Previous lncRNAs profile studies have demonstrated aberrant expression of a series of lncRNAs, including taurine upregulated gene 1 (TUG1), in ICC specimens [5]. TUG1 is a poorly conserved, long intergenic noncoding RNA that has been reported to regulate several genes involved in cell proliferation, differentiation, and invasion [6–8]. TUG1 has been shown to be regulated by Notch in glioma [9], and by p53 in non-small cell lung cancer [10]. To date, however, the clinical significance and specific role of TUG1 in ICC has not been investigated.

Numerous reports have shown that in addition to high glycolytic rates, increased glutamine metabolism plays a key role in the progression of many tumors [11]. Interestingly, several lncRNAs have been shown to contribute to metabolic dysregulation in cancer. Among them are CCAT2, which was shown to regulate glutamine metabolism in an allele-specific manner [12], and UCA1, which was shown to regulate the expression of glutaminase 2 (GLS2) through interaction with miR-16 [13]. Whether TUG1 promotes ICC progression through metabolism reprogramming has not, however, been yet defined.

The current study addressed the prognostic value of TUG1 upregulation in ICC and, through *in vitro* and *in vivo* experiments, the mechanisms underlying TUG1’s contribution to ICC development and progression. We found that TUG1 acts as a competing endogenous RNA (ceRNA) that binds miR-145 and prevents downregulation of Sirt3 and GDH, leading to aberrant glutamine metabolism and ICC progression. Our results might offer a potential therapeutic target for ICC treatment.

## RESULTS

### TUG1 expression in ICC and association with clinical features

**Figure 1:**
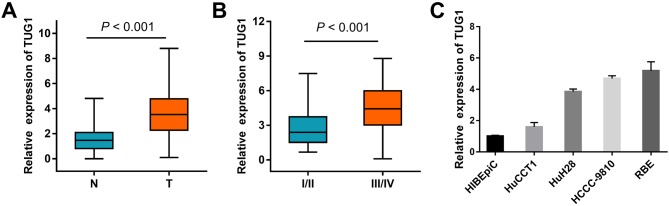
TUG1 expression in ICC clinical samples **(A)** Relative expression of TUG1 in ICC specimens compared with patient-matched non-tumor samples. GAPDH was used as endogenous control. **(B)** Correlation between TUG1 expression and tumor stage. The expression of TUG1 in I+II/III+IV stage tumors was normalized to that of the corresponding paired normal tissues. **(C)** Expression of TUG1 in human ICC cell lines, compared to HIBEpiC cells. Data are presented as mean ± SD, and *P* values were calculated by Student’s t-test.

The expression of TUG1 was determined by qRT-PCR in paired ICC and normal tissue samples from 102 patients. As shown in Figure 1A, TUG1 was overexpressed in ICC compared to corresponding non-tumor tissues (3.68 ± 0.2 vs 1.54 ± 0.1, *P* < 0.001). We next analyzed the relationship between TUG1 expression and clinical features of ICC. High TUG1 expression was significantly associated with tumor stage (*P* < 0.001, Figure 1B), intrahepatic metastasis (*P* = 0.001), lymph node metastasis (*P* < 0.001), and perineural invasion (*P* = 0.029). There was no association between TUG1 expression and age, sex, tumor size, CA199, differentiation, hepatitis B/C infection, or venous invasion (Supplementary Table 1). TUG1 was also highly expressed in ICC cell lines compared to normal biliary epithelium cell (HIBEpiC) (Figure 1C).

### Upregulation of TUG1 is associated with poor prognosis in ICC patients

**Figure 2:**
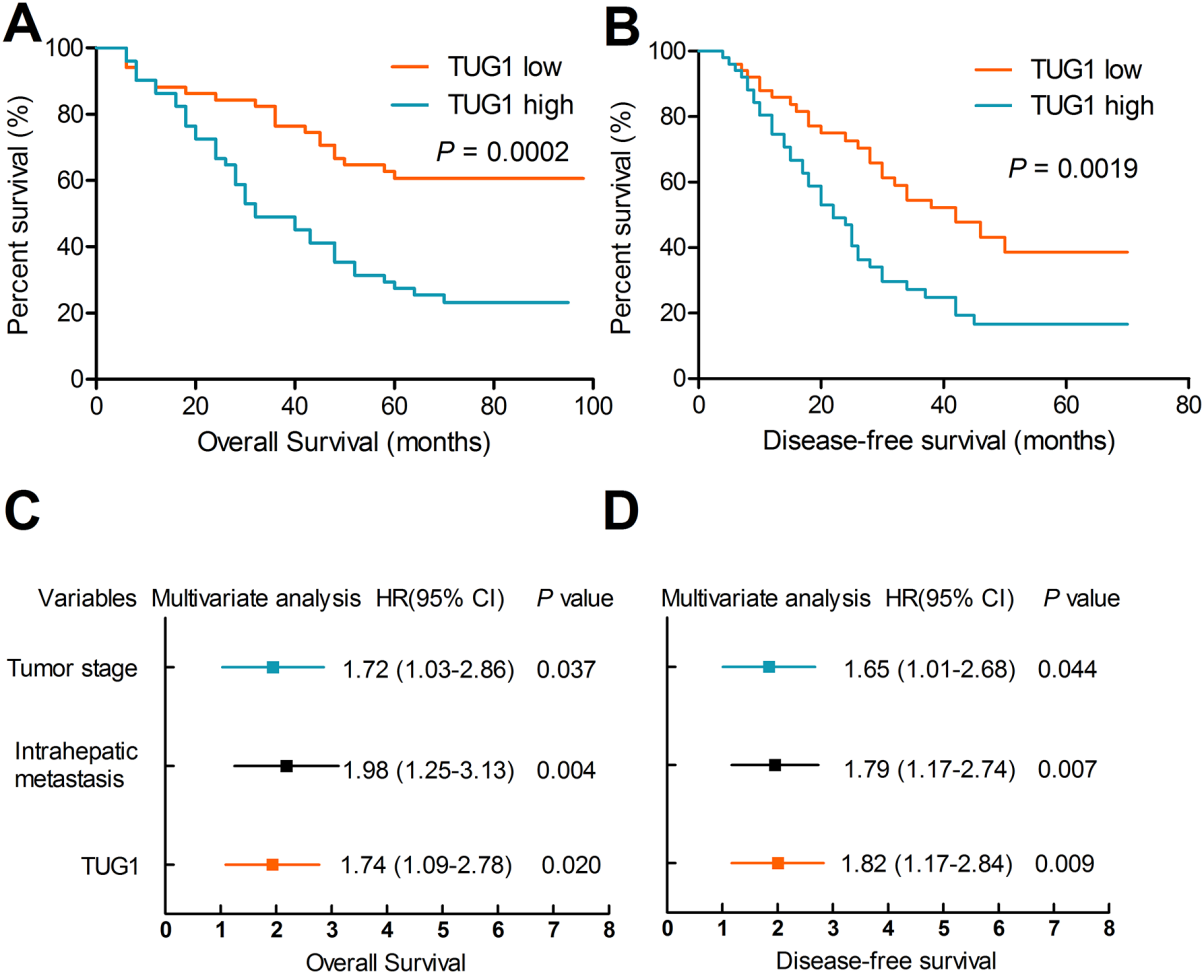
Upregulation of TUG1 is associated with poor prognosis Patients with high TUG1 expression had poorer overall survival **(A)** and poorer disease-free survival rates **(B)** than patients with low TUG1 expression. **(C-D)** TUG1 expression, tumor stage and intrahepatic metastasis were independent prognostic indicators for both OS and DFS.

To explore the clinical significance of TUG1, we attempted to assess the correlation between TUG1 expression and clinicopathological factors. The median ratio of relative TUG1 expression (3.78) in tumor tissues was used to divide the samples into two groups. Kaplan-Meier survival analysis and the log-rank test showed that patients with high TUG1 expression had significantly decreased overall survival (OS) (27.3% vs. 57.9%; HR 2.44; 95% CI 1.57-3.78; *P* < 0.001), and disease-free survival (DFS) (HR 2.04; 95% CI 1.37-3.04; *P* < 0.001) compared with patients with low TUG1 expression (Figures 2A-2B). Univariate analysis revealed that tumor stage, intrahepatic metastasis, venous invasion, perineural invasion, and TUG1 expression were all significantly correlated with OS and DFS (Table 1). Furthermore, multivariate analyses indicated that TUG1 expression, tumor stage, and intrahepatic metastasis were independent prognostic factors for both OS and DFS (Figures 2C-2D).

**Table 1:** Univariate and multivariable Cox regression analyses for overall and disease-free survival

Factor	Univariate analysis				Multivariate analysis			
	HR	95% CI		*P*-value	HR	95% CI		*P*-value
**Overall survival**								
Tumor stage	2.50	1.56	4.01	<0.001^*^	1.72	1.03	2.86	0.037^*^
Intrahepatic metastasis	2.57	1.67	3.96	<0.001^*^	1.98	1.25	3.13	0.004^*^
Venous invasion	1.86	1.22	3.80	0.005^*^	1.18	0.75	1.88	0.472
Perineural invasion	1.59	1.04	2.44	0.031^*^	1.30	0.83	2.04	0.247
TUG1	2.44	1.57	3.78	<0.001^*^	1.74	1.09	2.78	0.020^*^
**Disease-free survival**								
Tumor stage	2.91	1.89	4.50	<0.001^*^	1.65	1.01	2.68	0.044^*^
Intrahepatic metastasis	2.25	1.52	3.34	<0.001^*^	1.79	1.17	2.74	0.007^*^
Venous invasion	1.74	1.16	2.61	0.007^*^	1.12	0.72	1.73	0.626
Perineural invasion	1.62	1.09	2.39	0.016^*^	1.33	0.88	2.00	0.178
TUG1	2.04	1.37	3.04	<0.001^*^	1.82	1.17	2.84	0.009^*^

*HR* relative risk, *95% CI* 95% confidence interval.

^*^ Statistically significant *P*<0.05, Cox proportional hazard regression model.

### TUG1 knockdown reduces ICC cell proliferation, migration, and invasion

**Figure 3:**
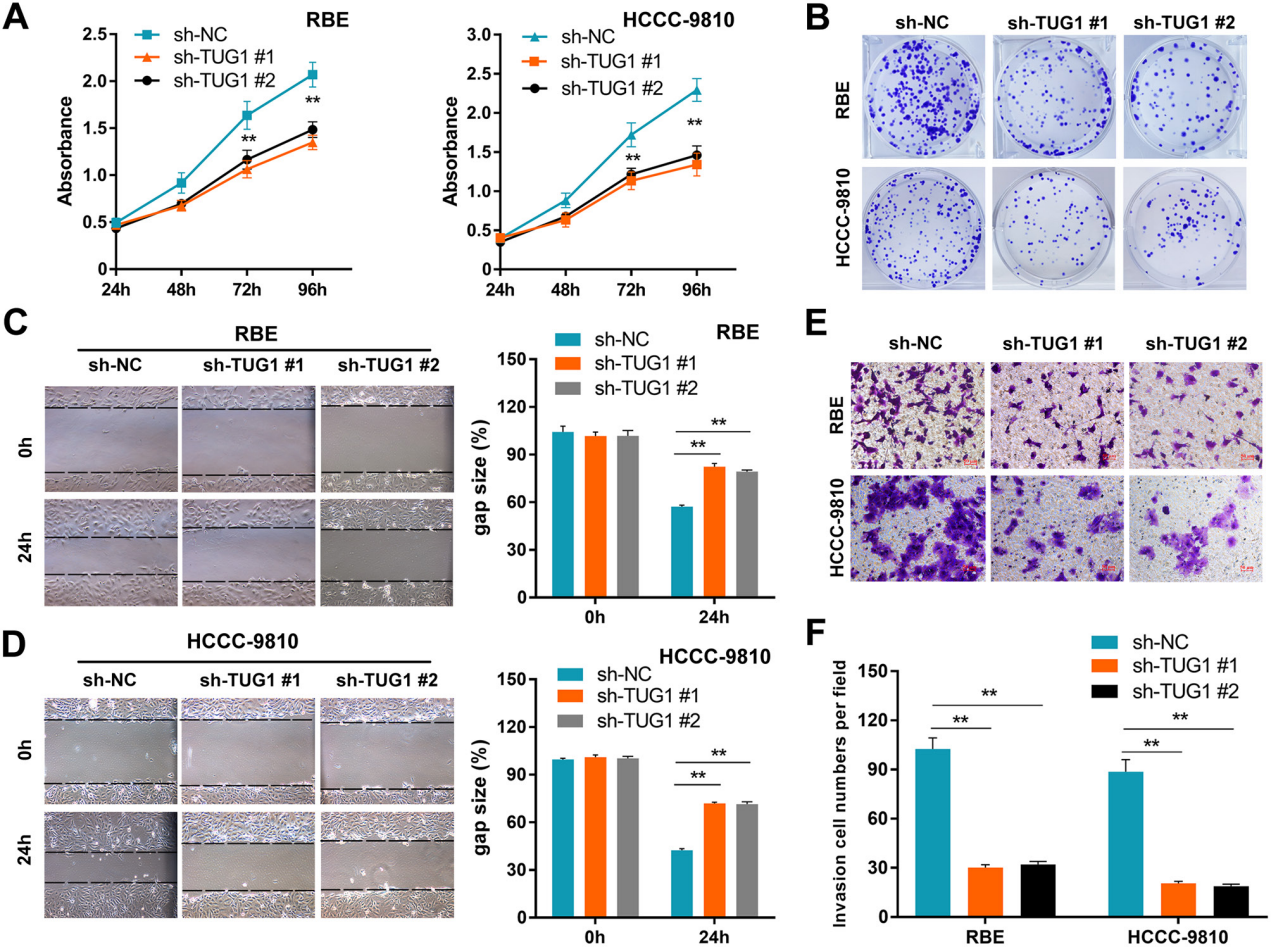
TUG1 knockdown impairs ICC cell proliferation and motility Effects of TUG1 knockdown on cell proliferation **(A)**, colony formation **(B)**, migration **(C-D)**, and invasion **(E-F)** in ICC cells. Data are presented as mean ± SD based on three independent experiments. ^**^P < 0.01 compared to control (Student’s t-test).

Although the tumor-promoting capacity of TUG1 has been well documented in other cancers [14], its potential contribution to ICC pathogenicity remains undefined. To address this issue, ICC cells were transfected with sh-TUG1 or sh-NC plasmids to construct stably transfected cell lines. Then, the efficiency of TUG1 knockdown was validated by qRT-PCR analysis (Supplementary Figure 1). MTT assays showed that TUG1 knockdown significantly suppressed cell proliferation (Figure 3A). Consistently, colony formation assays demonstrated that the clonogenic ability was also significantly decreased after TUG1 knockdown (Figure 3B). We next investigated the effect of TUG1 knockdown on cell migration and invasion by performing wound-healing and transwell assays. Wound healing assay showed that TUG1 knockdown caused a marked decrease in cell motility (Figures 3C-3D). Similarly, transwell invasion assay demonstrated that TUG1 knockdown led to a significant reduction in the invasive capacity of ICC cells (Figures 3E-3F).

### TUG1 knockdown suppresses tumor growth *in vivo*

**Figure 4:**
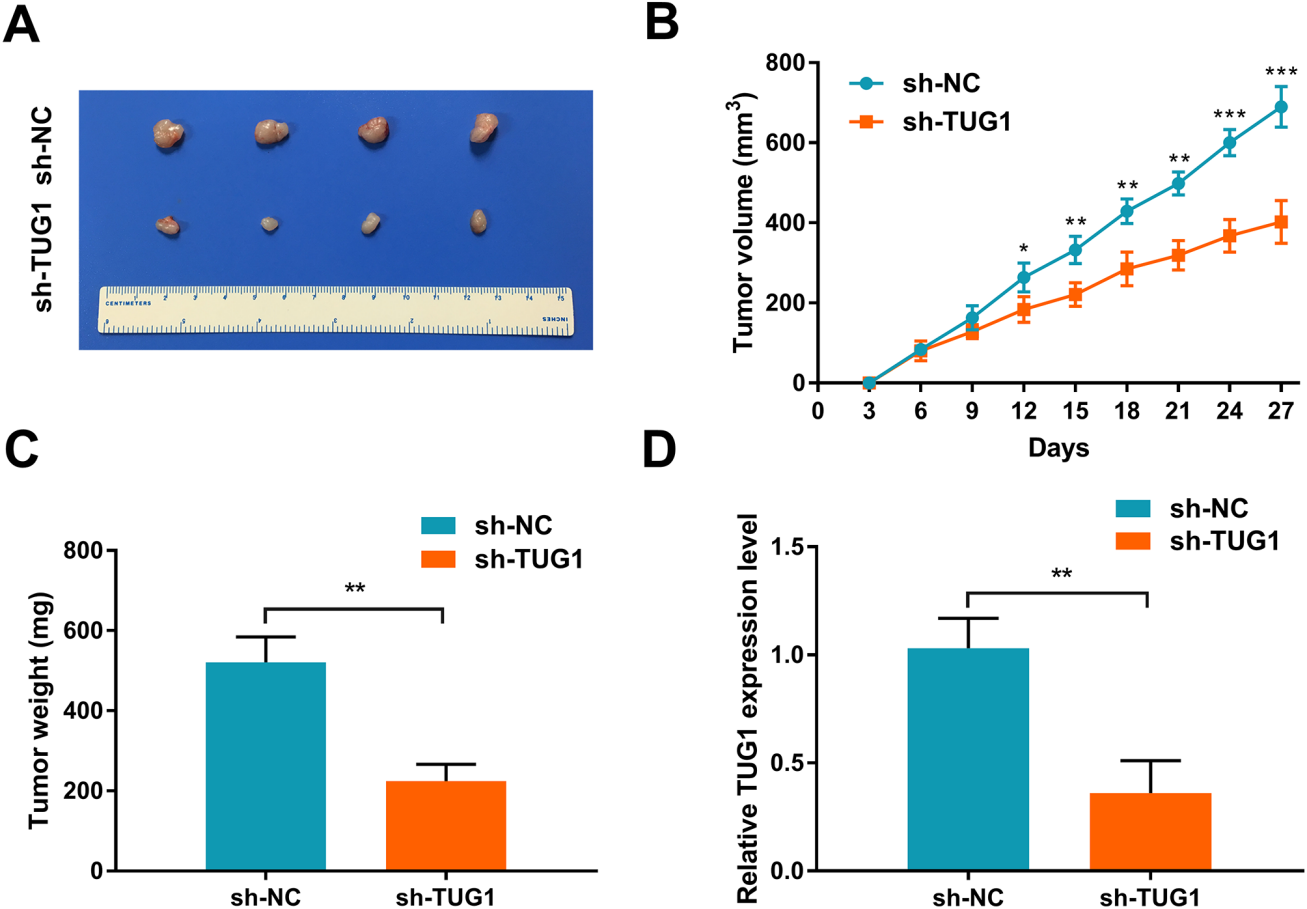
TUG1 knockdown suppresses tumor growth *in vivo* **(A)** Effect of TUG1 knockdown on tumor growth in a xenograft nude mice model (n = 4 per group). **(B)** Growth curves of xenograft tumors after subcutaneous injection with shTUG1 and control cells. Tumor volumes were measured every 3 days after inoculation. **(C)** Tumor weights were measured after mice sacrificed. **(D)** TUG1 expression in xenograft tumors was detected by qRT-PCR. Data represent the mean ± SD of three independent experiments. ^**^P < 0.01 compared to control (Student’s t-test).

Next, we investigated the effect of TUG1 inhibition *in vivo* using a xenograft nude mice model. To this end, 5×10^6^ sh-TUG1 RBE cells or control cells were injected subcutaneously into the right dorsal flanks of nude mice. We found that knockdown of TUG1 markedly reduced tumor growth compared with the control group (Figure 4A). Moreover, both tumor volume and weight were dramatically reduced in the sh-TUG1 group compared with the control group (Figures 4B-4C), together with the down-regulation of TUG1 expression (Figure 4D). Collectively, these findings suggest that TUG1 acts as an oncogene in ICC.

### TUG1 negatively regulates the expression of miR-145

**Figure 5:**
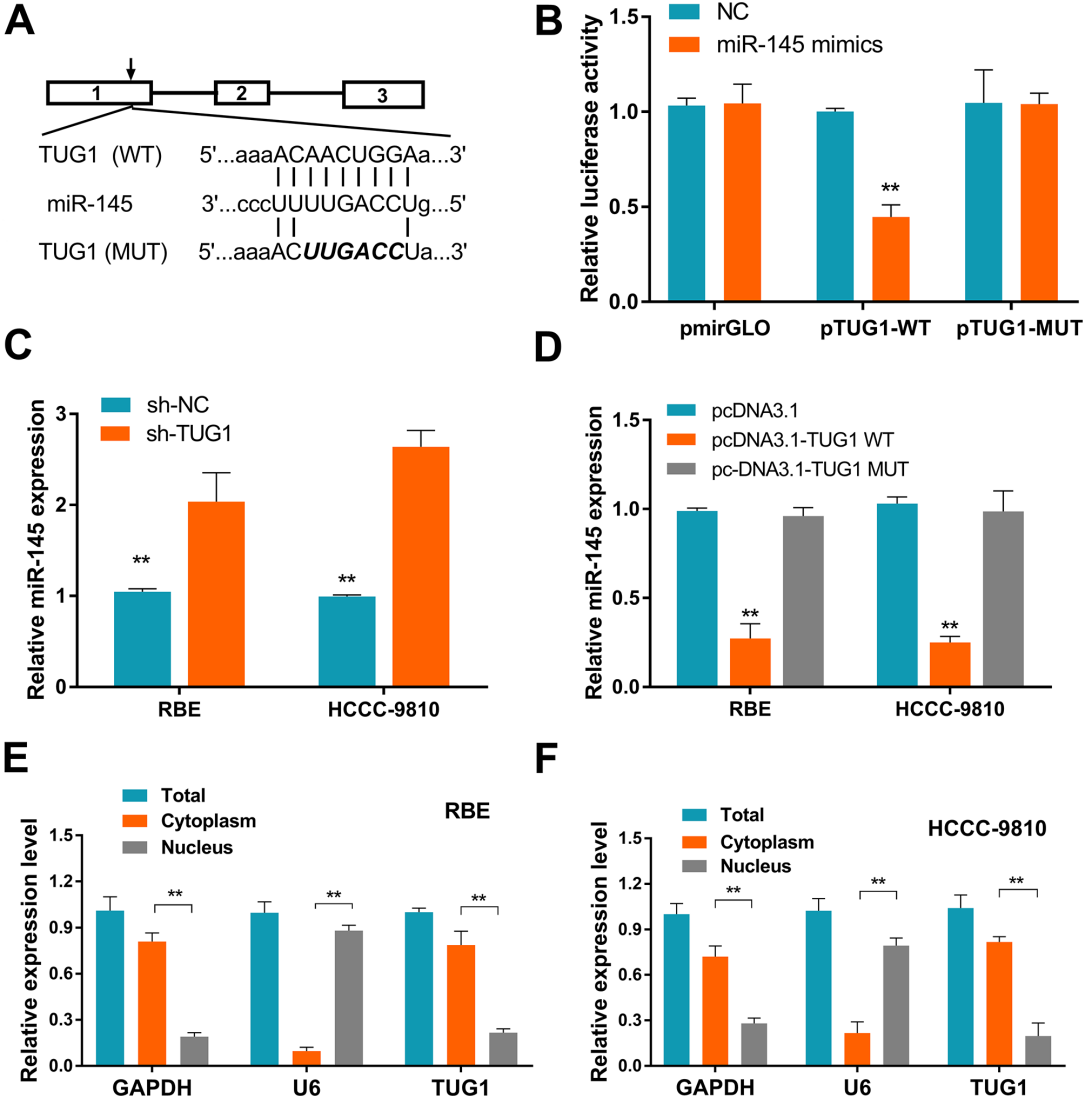
Identification of miR-145 as a target of TUG1 **(A)** Putative binding sites of miR-145 in TUG1 sequence, identified using starBase v2.0. **(B)** Luciferase activity in cells co-transfected with miR-145 mimics and luciferase reporters containing TUG1 or mutant transcripts. Data are presented as the relative ratio of firefly/Renilla luciferase activity. Results shown are from three independent experiments. **(C)** Effect of TUG1 knockdown on the expression of miR-145 in ICC cells. **(D)** Effect of TUG1 overexpression on the expression of miR-145 in ICC cells. **(E-F)** TUG1 localization (%) in the cytoplasmic and nuclear fractions of ICC cells. GAPDH and U6 served as cytoplasmic and nuclear localization markers, respectively. Data are presented as the mean ± SD of triplicate experiments. ^**^P < 0.01 compared to control (Student’s t-test).

Recent evidence has shown that lncRNAs not only exert their function by regulating protein expression, but also act as ceRNAs by binding to miRNAs and inhibiting their activity [15]. To assess whether TUG1 could function as a ceRNA, the bioinformatics databases DIANA-LncBase and starBase v2.0 were examined to search for potential TUG1 targets. Among the potential miRNAs, miR-145 was the most downregulated in ICC as previous study reported [16, 17]. The putative miR-145 binding site of TUG1 was shown in Figure 5A. Therefore, we performed a dual-luciferase reporter assay to confirm the direct binding between TUG1 and miR-145. We found that miR-145 mimics significantly reduced the luciferase activities of wild-type TUG1 reporter vector. However, co-transfection with miR-145 mimics and mutated TUG1 had no effect on the luciferase activity compared to that of control cells (Figure 5B). Furthermore, transfection of sh-TUG1 markedly increased the expression level of miR-145 in ICC cells (Figure 5C). While transfection with wild-type TUG1 clone suppressed the expression level of miR-145 in ICC cells (Figure 5D). In addition, qRT-PCR analysis of fractionated nuclear and cytoplasmic RNA revealed that TUG1 was preferentially located in the cytoplasm (Figures 5E-5F), providing prerequisite for proper interaction between TUG1 and miR-145.

Next, to study whether miR-145 was involved in the tumor-promoting capacity of TUG1. We have performed co-transfection studies and found that miR-145 inhibitors could rescue cell proliferation and migration caused by TUG1 depletion (Supplementary Figure 2). These results further indicated that TUG1 might exert its function partially through miR-145.

### TUG1 regulates glutamine metabolism in ICC cells

**Figure 6:**
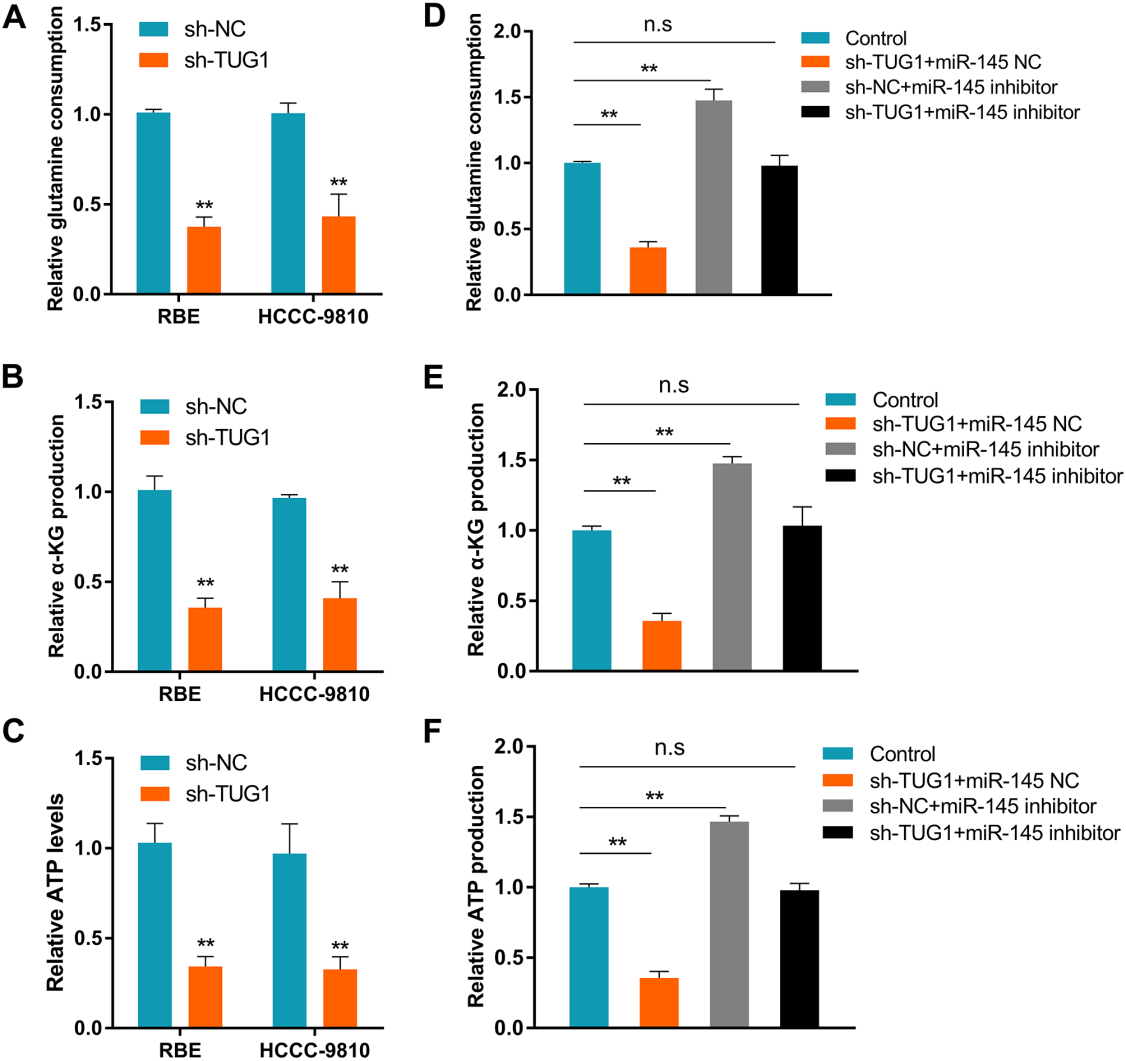
TUG1 increases glutamine metabolism in ICC by sponging miR-145 **(A)** Glutamine consumption, **(B)** α-KG production, and **(C)** cellular ATP levels in ICC cancer cells after TUG1 knockdown. **(D)** Glutamine consumption, **(E)** α-KG production, and **(F)** cellular ATP levels in ICC cancer cells transfected with sh-TUG1+miR-145 NC, sh-NC+miR-145 inhibitor, sh-TUG1+miR-145 inhibitor, and in control cells. Data are presented as the mean ± SD based on three independent experiments. ^**^P < 0.01 compared to control (Student’s t-test).

Aberrant energy metabolism is a critical hallmark of cancer [18]. Since previous study demonstrated that TUG1 could modulate mitochondrial bioenergetics [19], we asked whether TUG1 promotes ICC progression through metabolic reprogramming. Interestingly, transfection of sh-TUG1 caused a dramatic reduction in cellular glutamine consumption, α-KG production, and ATP levels in ICC cells (Figures 6A-6C), without affecting glucose consumption or lactate production (Supplementary Figure 3). To clarify whether miR-145 was involved in the reduction of glutamine metabolism elicited by TUG1 knockdown, co-transfection experiments were conducted in ICC cells. As shown in Figures 6D-6F, co-transfection of shNC + miR-145 inhibitor lead to increased glutamine consumption, α-KG production, and cellular ATP levels. Meanwhile, co-transfection of sh-TUG1+miR-145 inhibitor reversed the increase in glutamine consumption referred above. These results support the hypothesis that the TUG1/miR-145 interaction regulates glutamine metabolism in ICC cells.

### TUG1 regulates Sirt3 and GDH expression via miR-145

**Figure 7:**
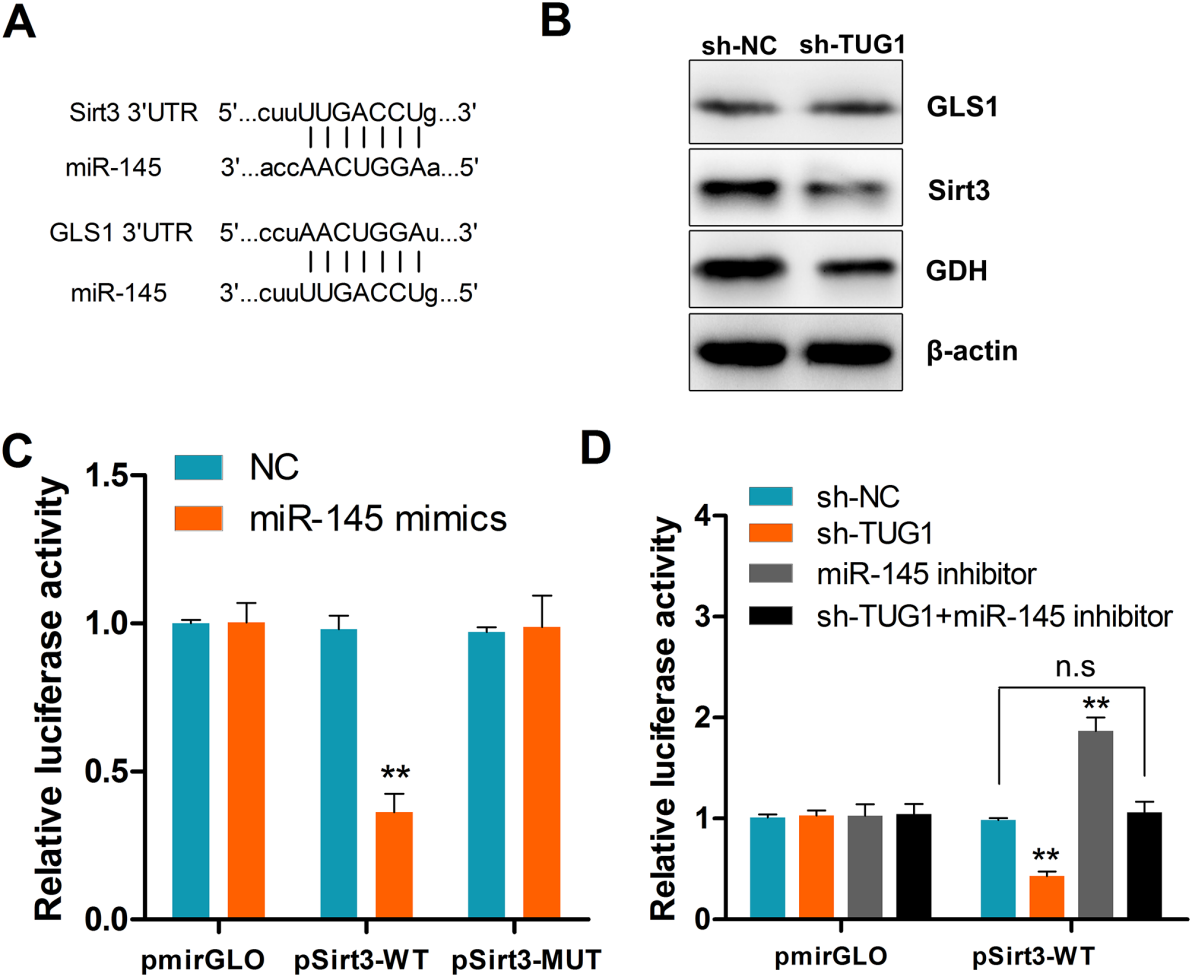
TUG1 upregulates Sirt3 and GDH expression by sequestering miR-145 **(A)** Schematic depiction of a miR-145 seed match in the 3’-UTR ofSirt3 and GLS1 mRNAs as identified by TargetScan. **(B)** Western blot assessment of Sirt3, GLS1, and GDH protein expression in shTUG1-transfected cells. GAPDH served as a loading control. **(C)** Luciferase activity in cells co-transfected with NC or miR-145 mimics, and luciferase reporters containing Sirt3 or mutant transcripts. **(D)** Luciferase activity in cells co-transfected with NC, sh-TUG1, miR-145 inhibitor, sh-TUG1+miR-145 inhibitor, and luciferase reporters containing Sirt3 or no specific promoter. Data are presented as mean ± SD based on three independent experiments. ^**^P < 0.01 compared to control (Student’s t-test); n.s, not significant.

The prominent role of miRNAs in regulating protein expression through posttranscriptional repression of mRNAs has been well established [20]. In view of the above results, we used the TargetScan algorithm to find out whether glutaminase (GLS), glutamate dehydrogenase (GDH), and/or their regulators are targets of miR-145. Results revealed Sirt3, a positive regulator of GDH [21], and glutaminase 1 (GLS1) as potential targets of miR-145 (Figure 7A). Transfection experiments showed that ectopic expression of miR-145 significantly reduced Sirt3 and GDH, but not GLS1 protein levels (Figure 7B). To confirm that Sirt3 is a direct target of miR-145, the full length 3’-UTR fragments of Sirt3 and corresponding mutant counterparts were cloned directly downstream of the firefly luciferase gene. As shown in Figure 7C, miR-145 transfection significantly suppressed the luciferase activity of the reporter with Sirt3 wild-type 3’-UTR. Moreover, this effect was reproduced by TUG1 knockdown, and could be rescued by co-transfection with miRNA-145 inhibitor (Figure 7D). These data confirmed that TUG1 antagonizes miR-145 and regulates the Sirt3/GDH axis.

To further study whether GDH was involved in miR-145 mediated glutamine metabolism. We performed co-transfection experiments, and found that overexpression of miR-145 could significantly inhibit glutamine metabolism, while ectopically expressed GDH can rescue the effect of miR-145 overexpression (Supplementary Figure 4).

## DISCUSSION

Numerous studies have reported the contribution of lncRNAs to cancer pathogenesis [22, 23]. TUG1, a highly conserved lncRNA, was first identified in a study addressing the differentiation of mouse retinal cells [7]. Recent evidence demonstrated that TUG1 has pro-tumorigenic actions in several cancer types. For instance, TUG1 expression is induced by Notch signaling in glioma stem cells, and suppresses neuronal differentiation-associated genes by binding to the polycomb repressive complex 2 (PRC2) [9]. TUG1 has also been shown to enhance tumor angiogenesis and VEGF expression through inhibition of miR-299 in glioblastoma cells [24], and to promote colorectal cancer cell proliferation and migration by induction of the epithelial-mesenchymal transition (EMT) process [25]. This oncogenic function of TUG1 has also been demonstrated in breast cancer [26], gastric cancer [27], and gallbladder carcinoma [28]. However, TUG1 has been shown to act as a tumor suppressor in non-small cell lung cancer, where TUG1 knockdown promoted tumor cell proliferation through epigenetic regulation of HOXB7 expression [10]. Although recent profile studies found that TUG1 is commonly upregulated in ICC [5], no studies have so far addressed TUG1’s function in this disease. We show here that TUG1 is upregulated in clinical ICC specimens, and high TUG1 expression correlates with poor survival and tumor progression. Importantly, our results revealed that TUG1 is an independent prognostic factor for survival, suggesting its potential usefulness in the clinical practice. Moreover, we demonstrated that TUG1 promotes ICC cell proliferation and motility by counteracting a miR-145-mediated reduction in glutamine metabolism. These results suggest that TUG1 has an oncogenic function in ICC.

Increasing evidence indicates that many lncRNAs contain miRNA binding sites and act as ‘competing endogenous RNAs’ (ceRNAs) that ‘sponge’ specific miRNAs and antagonize their functions [4, 29]. After conducting a bioinformatics analysis of TUG1’s predicted miRNA targets, we focused on miR-145 because it is the most prominently downregulated miRNA in ICC. Furthermore, TUG1 has been reported to trap miR-145 and downregulate its expression in several tumor types, stimulating cell proliferation and invasion in gastric cancer [27], promoting self-renewal in glioma stem cells by upregulation of SOX2 and c-Myc expression [9], and facilitating bladder cancer cell invasion by stimulating ZEB2-mediated EMT [30]. Our study presents strong evidence for a pro-tumoral role of the TUG1/miR-145 interaction in ICC and provides a plausible account of the mechanisms involved.

Aberrant energy metabolism is a critical hallmark of cancer [18]. Several studies have shown that in addition to abnormally high glycolysis, enhanced glutamine metabolism also fuels tumor progression and contributes to poor outcomes [31]. Whereas both oncogenes and tumor suppressors, among them mTORC1, K-RAS, and p53, are implicated in the turnover of cellular glutamine [32–34], several lncRNAs have, interestingly, been shown to affect metabolic fluxes in cancer cells. Hung *et al.* revealed that the androgen-induced prostate-specific lncRNA PCGEM1 regulates glycolysis by interacting with c-Myc to enhance its transactivation activity [35]. In gallbladder cancer, the lncRNA GCASPC regulates glycolysis by directly binding pyruvate carboxylase [36]. In ovarian cancer, the lncRNA NRCP interacts with STAT1 and RNA polymerase II, leading to increased expression of glucose-6-phosphate isomerase and modulation of glycolysis [37]. Recently, the lncRNA CCAT2 has been shown to regulate glutamine metabolism in an allele-specific manner by interacting with the CFIm complex, fine-tuning the alternative splicing of glutaminase [12]. Li *et al.* found that the lncRNA UCA1 regulates the expression of GLS2 by interfering with miR-16, and represses ROS formation in bladder cancer cells [13].

The present work provides evidence supporting a critical role for TUG1 in ICC progression by showing that this lncRNA contributes to increased glutamine metabolism and enhanced tumorigenic potential by antagonizing miR-145 and indirectly upregulating the expression of Sirt3 and GDH. Our results suggest that the lncRNA TUG1 might be a useful prognostic biomarker in ICC patients and also a novel therapeutic target to prevent or counteract metabolic reprogramming in ICC.

## MATERIALS AND METHODS

### Tissue samples and cell lines

Tumor samples and adjacent non-tumor tissues were obtained from patients who underwent radical tumor resection between January 2008 and December 2012 in Sun Yat-sen Memorial Hospital. ICC diagnosis was histopathologically confirmed. Informed consent was obtained before sample collection from the patients. The ICC cell lines HuH28, HuCCT1, RBE, and HCCC-9810, as well as normal intrahepatic biliary epithelial cells (HIBEpiC) were maintained in RPMI-1640 supplemented with 10% FBS (Gibco, Grand Island, USA). The cultures were maintained in a humidified 5% CO_2_ incubator at 37°C.

### RNA extraction and quantitative real-time PCR

Total RNA was extracted from cells or tissues with TRIzol reagent (Invitrogen, USA), and RNA purity was measured computing the A260/A280 ratio using a NanoDrop spectrophotometer. Total RNA was converted to cDNA by reverse transcription using oligo (dT) primers and SuperScript II Reverse Transcriptase (Invitrogen, USA). For quantitative real-time PCR (qRT-PCR), three replicates of each sample were amplified and analyzed with a Roche Light-Cycler (Roche, Basel, Switzerland). Relative transcript expression levels were calculated by the 2^-ΔΔ^CT method. Primers for qRT-PCR are shown in Supplementary Table 2.

### Cell transfection and generation of TUG1 knockdown cells

TUG1 and control (shNC) shRNA sequences were purchased from GenePharma (Shanghai, China). MiR-145 mimic, inhibitor, and the respective negative control (NC) were purchased from RiboBio (Guangzhou, China). The lncRNA-TUG1 cDNA was amplified and subcloned into a pcDNA3.1 vector (Invitrogen); the resulting plasmid was named pcDNA3.1-TUG1 WT. The Quik Change Site-Directed Mutagenesis kit (Stratagene) was used to produce mutations in miR-145 response elements and synthesize pcDNA3.1- TUG1 MUT. Sequences and primers are shown in Supplementary Table 2.

For retroviral packaging, 293T cells were co-transfected with transfer plasmids and retroviral packaging vectors. For transduction, cultured cells were incubated with virus-containing supernatant in the presence of 8 mg/ml polybrene. After 48 h, infected cells were selected for 72 h with puromycin (2 mg/ml) or hygromycin (200 mg/ml). MiR-145 mimic, inhibitor, and NC were transfected using Lipofectamine2000 (Invitrogen) or Lipofectamine™ RNAiMAX according to the manufacturer’s instructions.

### Western blot analysis

Cells were lysed with RIPA buffer (Beyotime Biotechnology, China). Briefly, equal amounts of proteins for each sample were separated by SDS-PAGE and then transferred to polyvinylidene fluoride (PVDF) membranes for immunoblotting. The membranes were blocked in 5% fat-free milk for 2 h at room temperature, washed 3 times, and incubated with the following primary antibodies: GLS1, Sirt3, GDH (Abcam), and β-actin (Boster). An ECL chemiluminescence kit (Pierce) was used to detect antigen/antibody complexes.

### Isolation of cytosolic and nuclear fractions

The PARIS Kit (Life Technologies) was used to separate the nuclear and cytosolic fractions of ICC cells. The extracted RNA was used for downstream experiments. The expression of TUG1 in the nuclear and cytoplasmic fractions was detected via qRT-PCR, with GAPDH and U6 serving as cytosolic and nuclear controls, respectively.

### Cell proliferation and colony formation assays

Cell proliferation was measured in ICC cells seeded in 96-well plates (1×10^3^ cells per well). At 24, 48, or 96 h, MTT (20 μl, 0.5 mg/ml) was added to each well for 4 h. Then the medium was removed and DMSO (100 μl) was added to each well. Absorbance was measured at 490 nm.

For the colony formation assay, cells were seeded in 6-well plates in media containing 10% FBS in a humidified atmosphere for 2 weeks. Cells were then fixed, stained with 0.1% crystal violet (Sigma, USA) for 15 min, and the number of stained colonies was counted under a microscope. All experiments were carried out in triplicate.

### Wound-healing and Matrigel invasion assays

In the wound-healing assay cells were seeded in 6-well plates, the monolayer was wounded with a 20 μl sterile pipette tip, and cells were allowed to migrate for 24h. The migration index was calculated as previously described [38].

To perform the Matrigel invasion assay, cells were suspended in 100 μl of FBS-free medium, and then seeded in Matrigel pre-coated chambers. Medium with FBS in the lower chamber was used as chemoattractant. After incubation for several hours, the inserts were stained with crystal violet, and the number of cells invading the membrane was counted in five random fields. All experiments were performed at least three times.

### Xenograft mouse model

Animal experiments were performed following the Guide for the Care and Use of Laboratory Animals, and approved by the Animal Care and Use Committee of the Sun Yat-sen University. RBE cells stably expressing shTUG1 or control vector were collected, resuspended in RPMI-1640 (5×10^6^ cells/200 μl), and injected subcutaneously in the dorsal flanks of 6-week-old nude male BALB/c mice. Tumor volume was evaluated every 3 days. Mice were sacrificed after 27 days, and tumors were collected for further studies.

### Luciferase reporter assay

TUG1 and Sirt3 wild-type 3’-UTR containing the putative binding sites for miR-145 were amplified and cloned into pmirGLO vector (Promega, USA). Fusion PCR was employed to amplify the mutant 3’-UTR. Cells were plated in 96-wells, incubated for 24h to 80% confluence, and then co-transfected with wild-type or mutated reporter plasmid. The Dual-Luciferase Reporter Assay System (Promega, USA) was employed to measure luciferase activities 48h after transfection. All experiments were carried out at least in triplicate. Primers are shown in Supplementary Table 2.

### Glucose and glutamine uptake assay

Cells were cultured in glucose-free medium for 24h and then incubated with high-glucose medium for 1h. A fluorescence-based glucose assay kit (BioVision, USA) was employed to measure intracellular glucose content according to the manufacturer’s protocol. Glutamine uptake assays were performed as detailed previously [39]. Briefly, cells were seeded in plates with complete medium, which was replaced by glutamine-free medium, and then incubated with or without reagents. Glutamine concentration in the media was then measured using a BioProfile analyzer (Nova Biomedical, USA).

### Alpha-KG, lactate, and ATP measurements

A fluorescence-based lactate assay kit (BioVision, USA) was employed to determine intracellular α-KG and lactate levels. An ATP bioluminescent assay kit (Sigma, USA) was employed to determine intracellular ATP concentrations. Cells were suspended in ultrapure water. The reaction was initiated by adding ATP enzyme mix to the cell suspension, and luminescence was recorded with a spectrofluorometer.

### Statistical analysis

Statistical analysis was performed using SPSS 16.0 software (Chicago, IL, USA). The chi-square test or fisher exact test were used for non-parametric variables, and the Student t test was applied for parametric variables. Differences in patient survival were assessed using the Kaplan-Meier method. Univariate and multivariate Cox regression analyses were performed to assess the relative risk for each factor. All tests were two-sided and *P* < 0.05 was considered statistically significant.

## SUPPLEMENTARY MATERIALS FIGURES AND TABLES



## Abbreviations

ICC: Intrahepatic cholangiocarcinoma
TUG1: Taurine upregulated gene 1
GDH: Glutamate dehydrogenase
OS: Overall survival
DFS: Disease-free survival
ceRNA: competing endogenous RNA
