# Supplementary material for: LncRNA TUG1 sponges miR-145 to promote cancer progression and regulate glutamine metabolism via Sirt3/GDH axis
Source: Oncotarget. 2017 Oct 19;8(69):113650–61. doi: 10.18632/oncotarget.21922 (PMC5768353; doi:10.18632/oncotarget.21922)
Supplement: Supplementary file 1 [file oncotarget-08-113650-s001.pdf]

LncRNA TUG1 sponges miR-145 to promote cancer progression and regulate glutamine metabolism via Sirt3/GDH axis

SUPPLEMENTARY MATERIALS

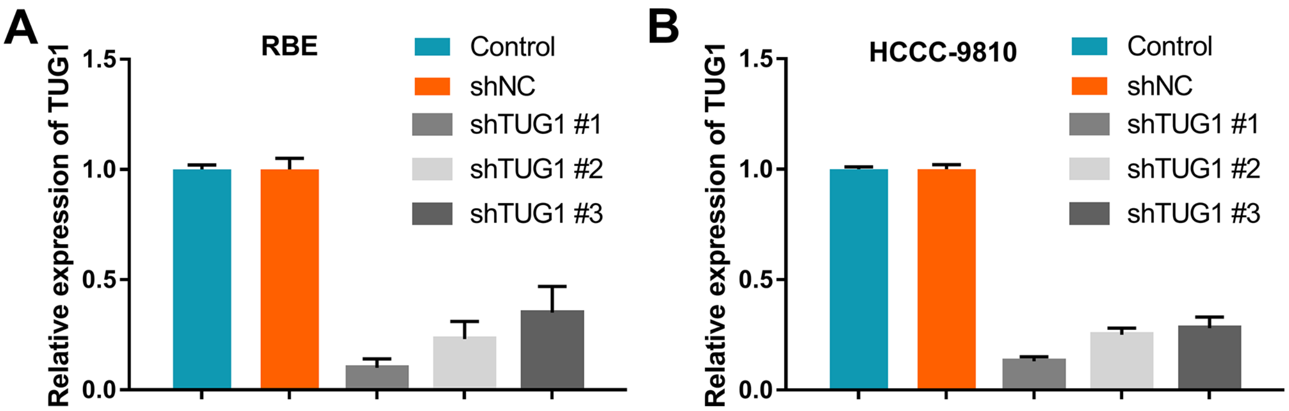

Supplementary Figure 1: Effect of sh-RNA on the expression of TUG1 in ICC cells.

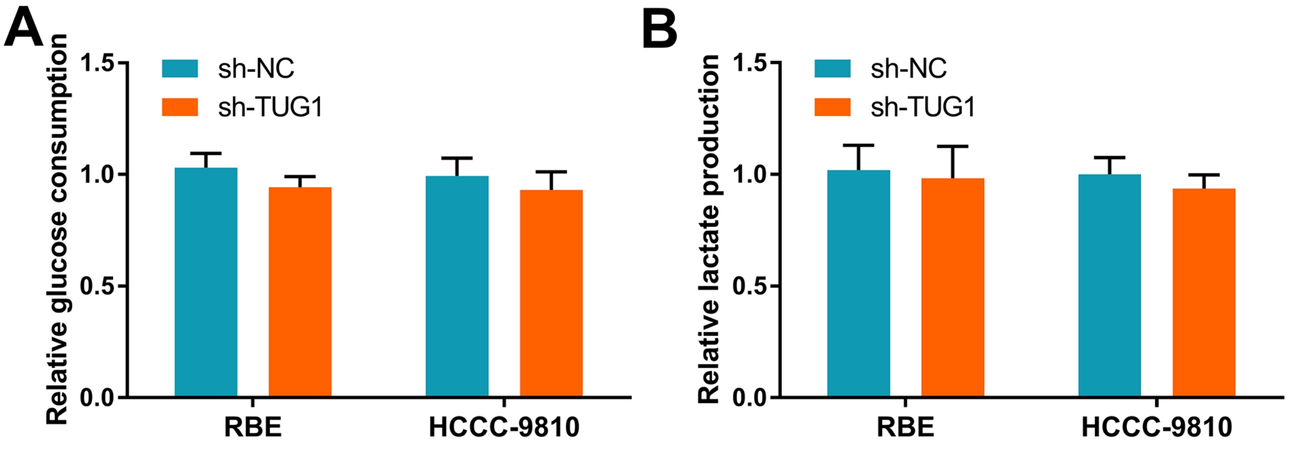

Supplementary Figure 2: Effect of TUG1 knockdown on glucose consumption and lactate production.

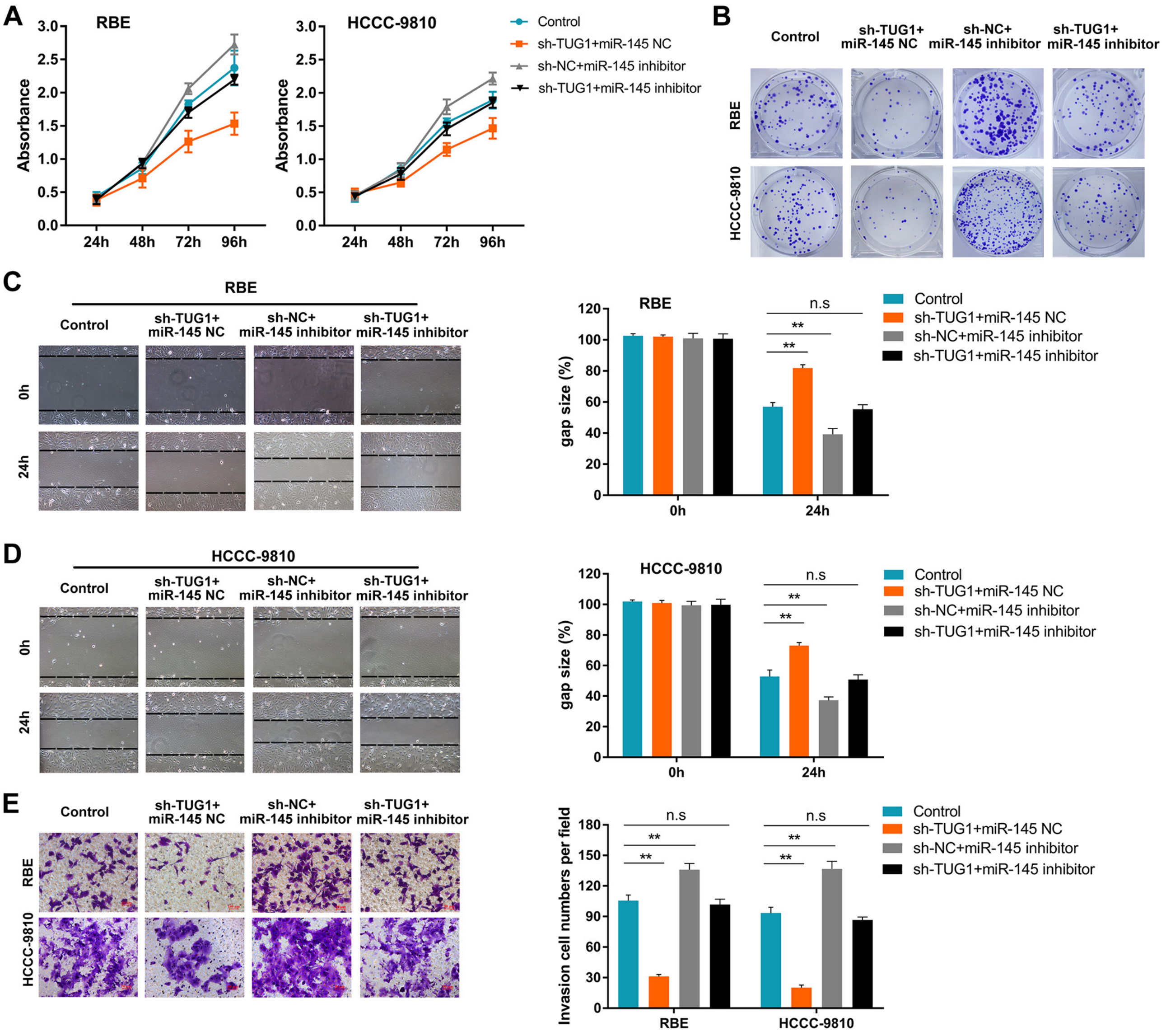

Supplementary Figure 3: TUG1 promotes ICC cell proliferation and migration partially through miR-145.

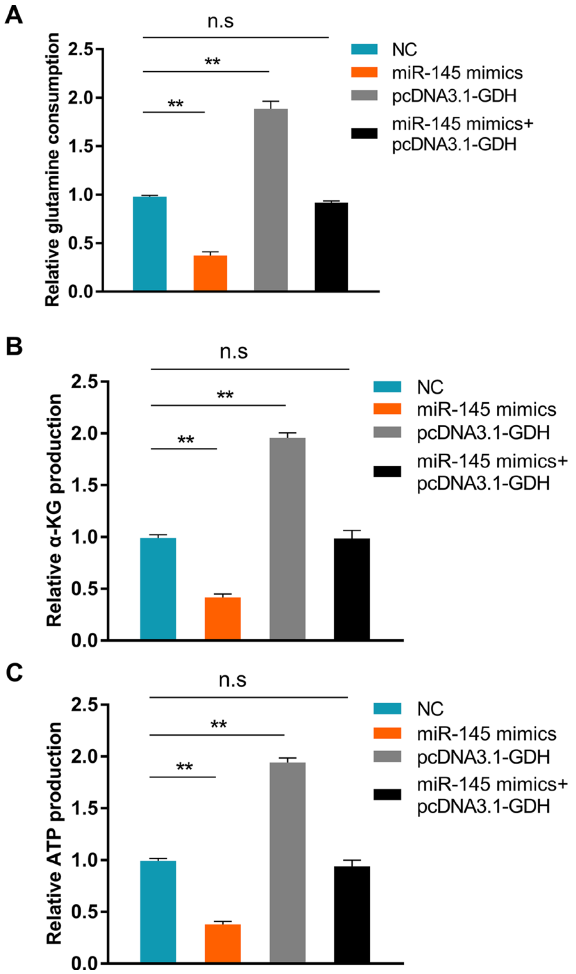

Supplementary Figure 4: GDH was involved in miR-145 mediated glutamine metabolism.

Supplementary Table 1: Clinicopathological factors and TUG1 expression in ICC patients

Characteristics		No.	TUG1		*P* value
			high group	low group	
Age	< 60	53	25	28	0.552
	≥ 60	49	26	23	
Sex	Male	60	31	29	0.687
	Female	42	20	22	
Tumor size	< 5cm	64	30	34	0.413
	≥ 5cm	38	21	17	
CA199	Normal	52	24	28	0.428
	Elevated	50	27	23	
Differentiation	Well	25	10	15	0.497
	Moderate	61	32	29	
	Poor	16	9	7	
Tumor stage	I/II	43	13	30	**<0.001***
	III/IV	59	38	21	
Hepatitis B/C	Negative	47	23	24	0.843
	Positive	55	28	27	
Intrahepatic metastasis	Negative	53	18	35	**0.001***
	Positive	49	33	16	
Lymph node metastasis	Negative	56	15	41	**<0.001***
	Positive	47	36	11	
Venous invasion	Negative	73	33	40	0.124
	Positive	29	18	11	
Perineural invasion	Negative	53	21	32	**0.029***
	Positive	49	30	19	

Supplementary Table 2: Sequences of RNA and DNA oligonucleotides

Name	Sense Strand (5' - 3')	Antisense Strand (5' - 3')
miR-145 mimics	GUCCAGUUUUCCCAGGAAUCCCU	GGAUUCCUGGGAAAACUGGACUU
mimics NC	UUCUCCGAACGUGUCACGUTT	ACGUGACACGUUCGGAGAATT
miR-145 inhibitor	AGGGAUUCCUGGGAAAACUGGAC	
inhibitor NC	CAGUACUUUUGUGUAGUACAA	
pri-miR-145	AACTCCAGCTGGTCCTTAG	TCTTGAACCCTCATCCTGT
TUG1	TAGCAGTTCCCCAATCCTTG	CACAAA TTCCCATCATTCCC
GDH	GGGATTCTAACTACCACTTGCTCA	AACTCTGCCGTGGGTACAAT
GLS1	GCTGTGCTCCATTGAAGTGA	GCAAACTGCCCTGAGAAGTC
Sirt3	CTTGGCGGCAGGGACGAT	CCCCGGCGATCTGAAGTCTG
GAPDH	AGCCACATCGCTCAGACAC	GCCCAATACGACCAAATCC
Tug1 3'-UTR WT	actgctagcCAAGTGGTACAGCCCTAAGC	acgtctagaAGGGTCTGGACAGAAACTCA
Tug1 3'-UTR MUT	AAATGTTGACCTACACAAAGCTTGA	AGTAGGTCAACATTTTTTTCCCTCC
Sirt3 3'-UTR WT	actgctagcCTGACAAGACCTCATGCCTG	acgtctagaCTCATGTCAACACCTGCAGT
Sirt3 3'-UTR MUT	AGACCTTGACCAATCCCAGACCGA	TGGGATAGGTCAAGGTCTGATTCA
TUG1 #1	5'-CACCGCGAGTCACTCTGTAATTTTTCA AGAGAAAATTACAGAGTGACTCGCTTTTT TG-3'	5'-GATCCAAAAAAGCGAGTCACTC TGTAATTTTCTCTTGAAAAATTACAGAG TGACTCGC-3'
TUG1 #2	5'-CACCGCCTTGTTTAGTGCATCTTTTTCAA GAGAAAAGATGCACTAAACAAGGTTTTT TG-3'	5'-GATCCAAAAAAGCCTTGTTTAGTGC ATCTTTTCTCTTGAAAAAGATGCACTAA ACAAGGC-3'
TUG1 #3	5'-CACCGCCATCATGATGTGGCCTTTTTCAA GAGAAAAGGVVACATCATGATGGTTTTTT G-3'	5'-GATCCAAAAAAGCCATCATGATGTG GCCTTTCTCTTGAAAAAAGGVVACATC ATGATGGC-3'
Control	5'-CACCGTTCTCCGAACGTGTCACGTCAAGA GATTACGTGACACGTTCGGAGAATTTTTTG-3'	5'-GATCCAAAAAATTCTCCGAACGTG TCACGTAATCTCTTGACGTGACACGT TCGGAGAAC-3'
